# Perineal urethrostomy to treat obstructive urolithiasis in a captive hand-raised steenbok (*Raphicerus campestris*)

**DOI:** 10.4102/jsava.v88i0.1536

**Published:** 2017-12-08

**Authors:** Luke A. Poore, Ross Hendry, Johan Steyl, Silke Pfitzer

**Affiliations:** 1 Department of Companion Animal Clinical Studies, University of Pretoria, South Africa; 2 Department of Paraclinical Sciences, University of Pretoria, South Africa; 3 Department of Production Animal Studies, University of Pretoria, South Africa

## Abstract

The steenbok (*Raphicerus campestris*) is a small antelope of the family Bovidae native to the African continent. Urolithiasis, the formation of urinary calculi in the urinary tract, can be caused by a variety of factors such as diet, dehydration, infection and anatomical predisposition. Urolithiasis, with uroliths identified as magnesium calcium phosphate carbonate in composition, was diagnosed in a hand-reared 5-month-old steenbok. Perineal urethrostomy was performed as a component of the broad treatment regime that included fluid therapy, antibiotic and anti-inflammatory treatment. However, the animal died 4 days later as a result of systemic hypoxia and energy depletion because of stress and cachexia. The challenges of post-surgical treatment, the importance of positive energy balance in small ruminants under stressful circumstances, as well as the role of diet of hand-reared antelope in predisposition to urolith formation are highlighted.

## Introduction

Clinical signs of urolithiasis vary according to the extent of urinary system compromise and may include dysuria, stranguria and haematuria (Van Metre et al. 1996). In chronic cases, prolonged accumulation of urine in the bladder may result in hydronephrosis, metabolic acidosis, hyperkalaemia, azotaemia and bladder rupture (Videla & Van Amstel 2016). Urolithiasis can be caused by dietary factors, dehydration, urinary tract infections and anatomical predisposition (Bailey 1981). Feedstuffs with a high phosphorus:calcium ratio have also been shown to predispose small ruminants to urolithiasis (Van Weeren, Klein & Voorhout 1987). The steenbok (*Raphicerus campestris*) under natural circumstances is preferentially a browser of small shrubs and receives the majority of its water from diet (Huntley 1972; Skinner & Chimimba 2005; Toit 1993). There are no previous published reports of urolithiasis in steenbok although urolithiasis in a hand-reared steenbok has been reported (S. Pfitzer [University of Pretoria] pers. comm., 09 April 2015). Urolithiasis has been reported in various antelope species in personal communications from zoological establishments (National Zoological Gardens of South Africa, Pretoria and Johannesburg Zoo, 2000). The Minnesota Urolith Centre, USA, has analysed uroliths in red forest duiker (*Cephalophus natalensis*), a species of antelope from central Africa similar to steenbok. Analysis of this total sample (*n* = 4) identified 25% struvite, 25% purine, 25% magnesium calcium phosphate carbonate and 25% mixed uroliths (Osborne et al. 2009a). A comparative study of urolith samples in captive African antelope species, including impala (*Aepyceros melampus*), gemsbok (*Oryx gazella*) and greater kudu (*Tragelaphus strepsiceros*), revealed 28.5% of the total sample (*n* = 11) to be calcium phosphate uroliths, 14% to be purine uroliths, 14% to be magnesium calcium phosphate uroliths and 43.5% other forms of uroliths (Osborne et al. 2009a).

## Ethical considerations

The owners of the steenbok described in the present case gave their informed consent for publication.

## Case presentation

A hand-reared 5-month-old male intact steenbok weighing 5 kg was presented at the Onderstepoort Veterinary Academic Hospital (OVAH) for investigation of dysuria of approximately 7 days’ duration. The steenbok had been maintained on a diet of commercially available wildlife pellets (Antelope 16% pellets, EPOL, Worcester, South Africa), lucerne hay, cow’s milk and fresh grass since weaning. The owner reported that diarrhoea had been present for 4 days prior to presentation and oral treatment with electrolyte solution had been undertaken. The steenbok had also been vocalising and straining during urination and had only managed to pass small amounts of urine for 5 days prior to presentation. On clinical examination at admission, the steenbok appeared to be agitated and a significantly enlarged bladder was found on palpation of the abdomen. No other abnormalities were evident on a general physical examination.

**FIGURE 1:**
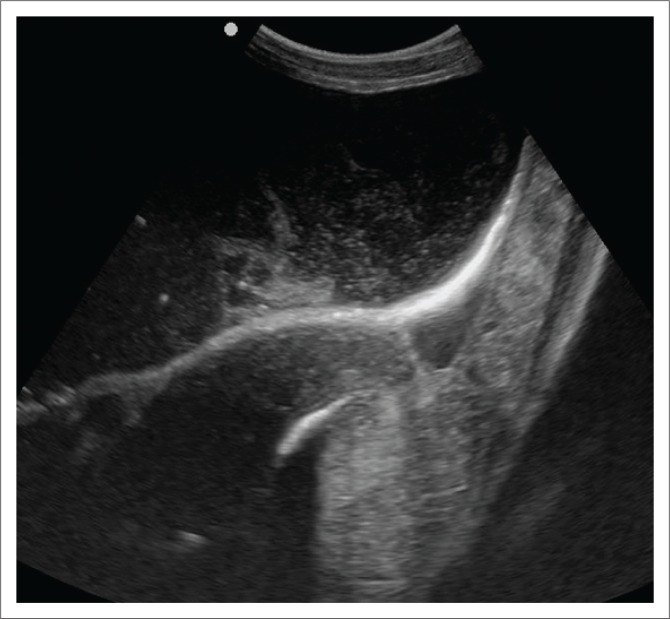
An ultrasonographic evaluation of the steenbok abdomen at presentation revealing distension of the bladder.

**FIGURE 2:**
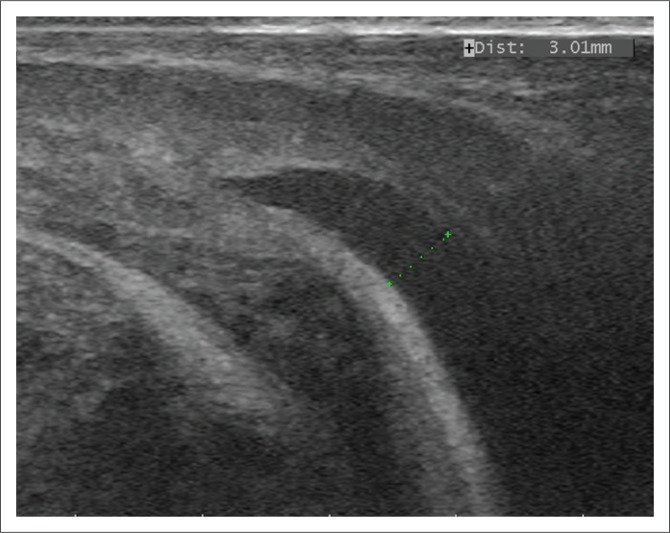
An ultrasonographic evaluation of the distal urethra showing a hyperechoic mass obstruction, 3.75 mm in diameter, proximal to the glans penis.

Urinalysis revealed urine with a pH of 8, and bacterial cocci, epithelial cells and small crystals were found on cytological examination. Urolithiasis was suspected and radiographic and ultrasonographic evaluations of the caudal abdomen performed. Radiographic examination of the abdomen was unremarkable, but the ultrasonographic evaluation revealed an enlarged bladder with fine hyperechoic debris on the ventral bladder wall. Agitation of the bladder by manipulation of the patient produced a snow-globe effect (Figure 1). Additionally, a hyperechoic mass, 3.75 mm in diameter, was observed obstructing the distal urethra proximal to the glans penis (Figure 2).

A provisional diagnosis of obstructive urolithiasis of the distal urethra was made. Treatment options considered included a tube cystotomy with normograde catheterisation or urine diversion surgery such as a perineal urethrostomy. The latter approach was decided upon in this case.

Haematological and biochemical analyses were found to be within normal limits for antelopes with a haematocrit of 49.4% (Pospísil et al. 1984; Vahala & Kase 1993). Pre-operative medication included amoxicillin clavulanic acid (10.5 mg/kg bodyweight [bwt]: Augmentin, Glaxo Smith Kline, Johannesburg, South Africa), meloxicam (2 mg/kg bwt: Metacam, Boehringer Ingelheim, Randburg, South Africa), hyoscine butyl bromide (4 mg/kg bwt: Buscopan, Boehringer Ingelheim, Randburg, South Africa) and butorphanol tartrate (0.05 mg/kg bwt: Torbugesic, Zoetis, Sandton, South Africa). After placement of an intravenous catheter in the left jugular vein, general anaesthesia was induced using propofol (1 mg/kg bwt: Propofol, Fresenius Kabi, Midrand, South Africa) and maintained with 3% isoflurane (15 mg/kg bwt: Isofor, Safeline Pharmaceuticals, Roodepoort, South Africa). The perineal region was aseptically prepared and draped. The urethral process at the distal urethra was amputated and an attempt made to perform retrograde catheterisation using a feline catheter (3 gauge Buster Barium Cat Catheter, Kruuse, Midrand, South Africa). Both catheter placement and retrograde lavage with ringers lactate (Hartmann’s solution, Fresenius Kabi, Midrand, South Africa) failed.

Perineal urethrostomy was performed by making a 3 cm mid-perineal incision ventral to the anus. The penile body was located through blunt and sharp dissection and freed from surrounding soft tissue. Dissection between the penile body and dorsal penile vessels was performed. Blunt dissection identified the retractor penis muscle and the ischiocavernosus muscle attachment to the penis. These were carefully transected before an incision was made through the corpus spongiosum into the urethra. The urethral mucosa was then left unsutured. A feline catheter (3 gauge Buster Barium Cat Catheter) was introduced into the urethra and directed initially towards the bladder. Lavage from this catheter did relieve the obstruction towards the glans penis but not proximally towards the bladder. An additional more proximal incision was made into the urethra, at which point retrograde lavage relieved the obstruction towards the bladder, with small white uroliths visible within the catheter. The feline catheter was then sutured into place facing towards the bladder and the urethrostomy site closed. Significant blood loss and hypothermia were evident during the surgical procedure, which lasted 2 h. The following day a peripheral venous blood sample was obtained for haematological analysis, which indicated a haematocrit of 23%. There are no published reference ranges for haematocrit values in the steenbok, although the normal haematocrit of the springbok (*Antidorcas marsupialis*), a larger antelope, has been recorded as 48% (Pospísil et al. 1984; Vahala & Kase 1993). This antelope was therefore anaemic. The steenbok was maintained on a therapeutic regimen of amoxicillin clavulanic acid (10.5 mg/kg bwt: Augmentin) and meloxicam (0.6 mg/kg bwt: Metacam) administered by subcutaneous injection. Oral fluid therapy via a bottle was commenced at 3-h intervals and ad lib water was supplied, as intravenous fluid therapy was not tolerated. The steenbok became agitated each time an attempt was made to install an intravenous fluid system. The oral fluids were supplemented three times daily with ammonium chloride (200 mg/kg bwt: Ammonium Chloride, Trans Pharm, Hermanstad, South Africa) to acidify the urine (Mavangira, Cornish & Angelos 2010; Sprake 2012). Acidification of the urine was important as struvite uroliths are most commonly associated with ruminants, the urine pH was increased to 8 and magnesium calcium phosphate crystals were present in the urine (Sullivan et al. 2013; Van Metre et al. 1996). The patient was given ad lib fresh buffalo thorn leaves (browse) and fresh grated vegetables at 0.5% bodyweight daily.

**FIGURE 3:**
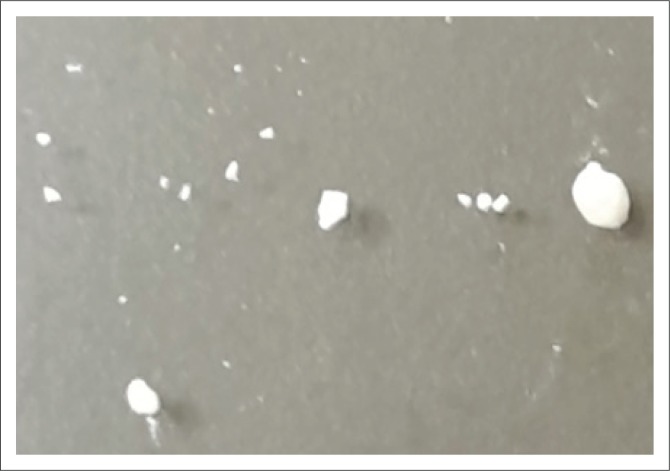
Macroscopic view of magnesium calcium phosphate carbonate uroliths obtained post-mortem from patient. Urolith size ranged from 1 mm to 6 mm in diameter.

The steenbok maintained a normal appetite, normal clinical parameters and regularly passed small amounts of urine through the catheter during the first 24 h post-operative period. The urine pH was 8 and specific gravity was 1.018. Magnesium calcium phosphate carbonate uroliths were found to be present in each urine sample (Figure 3).

During the next 24-h post-operative period, the steenbok maintained normal clinical parameters and appetite, although it started to vocalise and show straining behaviour when passing urine. Urinalysis at this stage revealed a pH of 7.5, small numbers of leukocytes and numerous erythrocytes in each urine sample.

The animal became depressed and showed a reduced appetite during the third day post-operatively. The indwelling urinary catheter was removed and the steenbok continued to pass small amounts of urine. Urinalysis revealed a pH of 6.4 and no crystals to be present.

The steenbok died at the beginning of the fourth post-operative day.

At necropsy, the carcass was anaemic, showed serous atrophy of cardiac coronary and bone marrow fat and multi-organ atrophy, all signs of cachexia. Dissection of the urogenital tract revealed severe post-surgical peri-urethral haematoma formation in the region of the urethrostomy site and a 7 mm unsutured linear incision in the ventral urethra, 1 cm distal to the ischiadic arch. Complete urethral obstruction with cream-white, sandy granules ranging between 0.5 mm and 1 mm in size, 50 mm proximal to the glans penis, was present. The urinary bladder was significantly distended but not ruptured and no macroscopic or microscopic ureteral and renal complications were seen. The liver showed mild periacinar hepatosis, which was microscopically characterised by moderate widespread periacinar hepatocellular necrosis. Other significant microscopic changes included localised chronic-active pyogranulomatous urethritis at the urethrostomy site and mild focal fibrosing cystitis. Significant enlargement of both adrenal glands was evident. Death was attributed to systemic hypoxaemia following significant post-surgical blood loss whilst suffering from anaemia and energy depletion because of stress and cachexia. No pathological evidence for uraemia was found.

Quantitative analysis of the uroliths was performed by the Minnesota Urolith Centre, USA. On physical appearance, the uroliths were 1.0 mm – 2.5 mm in diameter, had a rough surface and were white in colour (Figure 3).

The uroliths were classified as magnesium calcium phosphate carbonate uroliths.

## Discussion

The steenbok in this case report presented with magnesium calcium phosphate carbonate urolithiasis complicated by a concurrent urinary tract infection diagnosed by urinalysis and cytology. The coccoid bacteria seen on urinalysis could have been urease-producing, which would lead to a higher urine pH, predisposing the steenbok to urolith formation (Seaman & Bartges 2001). The high pH of the urine could also be attributed to the concurrent urinary tract infection, although it may have been because of secondary contamination, as urinary tract infection in male ruminants is rare (Yeruham et al. 2004). The formation of the magnesium calcium phosphate carbonate uroliths (Figure 3) was due to a combination of factors, including urinary tract infection, and an increased urine pH. Additionally, the level of calcium in the diet since weaning may have contributed to the urolith formation. Captive wildlife ruminants fed on a high concentrate diet, especially those like steenbok, which rely on their feed for hydration, should be given access to fresh vegetables such as carrots, cucumber and lettuce (Dierenfield 1997). These are high in moisture and low in both phosphorus and calcium. Diets high in oxalate, calcium and phosphorus should be avoided (Dierenfield 1997). The most effective way of reducing urolith formation is dietary management and effective environmental hygiene to limit urinary tract infections (Osborne et al. 2009b).

In our experience, small wild antelope such as steenbok, especially males, should be assessed for urolithiasis if presenting with signs of colic (S. Pfitzer [University of Pretoria] pers. comm., 09 April 2015). Urinalysis including sediment, crystal and urolith analysis is essential before effective medical treatment is decided upon (Lulich et al. 2016). Radiographic and ultrasonographic evaluation of suspected cases of urolithiasis has been suggested in small ruminants (Halland, House & George 2002; Kinsley et al. 2013; Van Weeren et al. 1987; Videla & Van Amstel 2016). Transabdominal ultrasonography has been shown to be valuable in assessing the kidneys, uroliths within the urinary system, the size of the bladder and the presence of free fluid in the abdomen, which could indicate bladder rupture (Ewoldt et al. 2006; Halland et al. 2002; Videla & Van Amstel 2016).

Plain radiographs have been recommended to assist in the diagnosis, surgical treatment and post-operative treatment of urolithiasis (Kinsley et al. 2013; Videla & Van Amstel 2016). In this case, we used plain radiographs in an attempt to localise the blockage and to assist in formulating a treatment protocol. Disadvantages of radiographic evaluation of urolithiasis cases include time, cost and difficulty in visualising some forms of urolith (Videla & Van Amstel 2016).

The surgical approach in this case initially utilised urethral process amputation, which is recommended as part of all treatment regimens because uroliths are often present in the process (Ewoldt et al. 2006; Videla & Van Amstel 2016). However, this technique has been shown to be effective in only 50% of caprine cases, with a recurrence rate of 80% – 90% within hours or days (Fortier et al. 2004). Placement of a catheter was then utilised to localise the blockage (May et al. 1998). Retrograde hydropulsion was also attempted but was unsuccessful. This technique has recently been reported as often being unsuccessful and has been shown to exacerbate damage to the urethra (Ewoldt et al. 2006; Videla & Van Amstel 2016). In this case, retrograde hydropulsion also prolonged the anaesthetic time, causing an additional stress to the steenbok and potentially decreasing the chance of survival post-operatively.

Percutaneous tube cystostomy is sometimes advocated in the treatment of urolithiasis but is primarily used as a cost saving procedure or when general anaesthesia is not available (Fazili et al. 2010; Kinjavdeker, Aithal & Pawde 2010; Singh, Gangwar & Devil 2014; Streeter, Washburn & McCauley 2002; Tamilmahan, Moshina & Karthik 2014). This surgical approach was not utilised in this case because of the high risk of complications and increased requirement for a second surgical procedure (Fortier et al. 2004; Videla & Van Amstel 2016).

Perineal urethrostomy, as used in this case, is a well-documented technique for the treatment of urolithiasis in small ruminants (Ewoldt et al. 2006; Haven et al. 1993; Videla & Van Amstel 2016). However, many authors consider a perineal urethrostomy to be a salvage procedure because of post-operative stricture formation (Tobias & Van Amstel 2013; Van Metre et al. 1996; Videla & Van Amstel 2016). Strictures have been reported to occur in 45% – 78% of cases (Haven et al. 1993; Tobias & Van Amstel 2013). Localised pyogranulomatous urethritis was evident at the urethrostomy site in this case when a post-mortem examination was performed and could have led to post-operative stricture formation. A superior skin to mucosa apposition could have been achieved if the urethral mucosa had been sutured to the skin on either side in this case and this may have prevented stricture formation if the steenbok had survived.

A modified proximal perineal urethrostomy has recently been described that may decrease the risk of post-operative urethral stricture formation and could have been utilised in this case to produce a better outcome (Tobias & Van Amstel 2013).

Haemorrhage after perineal urethrostomy and modified perineal urethrostomy has been reported as a common complication (Haven et al. 1993; Tobias & Van Amstel 2013). Possible causes include inadvertent transection of the dorsal penile artery, the internal pudendal artery, the deep artery of the penis and haemorrhage from transected muscles, fibrous or inflammatory tissue, or the corpus cavernosum and corpus spongiosum (Beckett, Reynolds & Hudson 1972; Tobias & Van Amstel 2013). In this case, surgical haemorrhage was most likely to have been from inadvertent transection of the dorsal penile artery. Ligation of the dorsal penile artery was not attempted in this case and has not been recommended because of the potential for avascular necrosis of the distal penile segment (Haven et al. 1993).

Medical treatment of magnesium calcium phosphate carbonate urolithiasis should consist of urine acidification, antibiotic treatment for urinary tract infection, fluid therapy and the provision of a diet with correct calcium:phosphate ratio (Ewoldt et al. 2006; Lulich et al. 2016; Tiruneh 2004). Urine acidification is important as calcium phosphate carbonate uroliths are known to precipitate in alkaline urine (Ewoldt, Jones & Miesner 2008). Broad spectrum antibiotic therapy is indicated to prevent or treat infection resulting from devitalised or inflamed urinary tissue and cavitational accumulation of urine (Ewoldt et al. 2008; Videla & Amstel 2016). Intravenous fluid therapy will help stabilise critical patients before surgical intervention and correct dehydration, reduce azotaemia and flush the urinary tract in the post-operative patient (Ewoldt et al. 2008). Lastly, the provision of a diet with correct calcium:phosphate ratio is important, with high calcium and low phosphorus concentration rations associated with calcium carbonate urolithiasis in goats (Nwaokorie et al. 2015). These medical treatments were adopted in this case.

Post-surgical management is particularly challenging for wildlife patients, especially non-hand-reared animals that need to be immobilised every time they are treated (Wallach & Boever 1993). The patient was moderately to severely anaemic from intra-operative blood loss as well as underlying cachexia. Blood transfusions in antelopes have been rarely reported, although successful xenotransfusion with bovine packed red cells in a Golden Wildebeest has been recently described (Buck et al. 2017). The duration of the surgical procedure and intra-operative hypothermia were additional stressful stimuli. These stimuli resulted in a post-surgical negative energy balance. Post-mortem examination showed the patient’s rumen to be filled with buffalo thorn leaves that were high in tannins, which would have reduced protein intake (Hay & Van Hoven 1988). The rumen of the steenbok in this case would also not have been adapted for buffalo thorn leaves, which could be another potential reason for inadequate energy intake. An antibiotic regimen of amoxicillin clavulanic acid was selected as it is excreted unchanged in urine, thus achieving high concentrations for a prolonged period and has a broad spectrum of antimicrobial activity (Sutherland, Croydon & Rolinson 1972). This antibiotic regimen might, however, have further limited the steenbok’s ability to effectively digest feed, by adversely affecting the balance of rumen flora (Tulstrup et al. 2015). The patient was also severely stressed, as shown by the enlarged adrenal glands found during post-mortem. Thus, the patient was in a negative post-surgical energy balance.

There are no previous reports of urolithiasis in the steenbok to the authors’ knowledge; this is also the first report of a perineal urethrostomy in a steenbok. The importance of energy balance in wildlife ruminant surgical patients is emphasised, and further research into nutritional support for wildlife ruminants is suggested. It is also hoped that improvements in our understanding of blood typing and coagulation screening of small antelopes will lead to improvements in pre-operative assessment and intra-operative management of surgical candidates.
